# Six Modern Plagues and How We Are Causing Them

**DOI:** 10.3201/eid1005.030936

**Published:** 2004-05

**Authors:** Dan Rutz

**Affiliations:** * Centers for Disease Control and Prevention, Atlanta, Georgia, USA

It’s not the price but rather the pathology of progress that author Mark Walters laments in Six Modern Plagues. Weaving anecdote with theory, Walters draws from his diverse backgrounds in veterinary medicine and journalism to link ecologic tampering to some of the most featured—if not feared—diseases of our time.

In recounting the origin of bovine spongiform encephalopathy (BSE) or “mad cow disease,” the author describes how, in compounding cattle feed with slaughterhouse byproducts, we converted our oldest domesticated herbivores into meat eaters. Bovine trickery aside, Walters renders his frank assessment that, “violating such evolutional boundaries can seem unnatural if not disgusting.” He goes on to add a damaging link to the food chain, leading to >100 human cases of always-fatal variant Creutzfeldt-Jackob disease (vCJD). Most cases occurred in or are related to the United Kingdom, where, by late 2000, more than 35,000 herd of cattle were infected with BSE. Though the practice of supplementing feed with animal byproducts has, for the most part, been abandoned, Walters suggests that certain risks remain, as prions, the subviral infectious agent responsible for mad cow and vCJD are also found in wild game, though, to date, no one has connected consumption of deer or elk meat with vCJD.

However, far from being out of the woods, humankind remains vulnerable to exotic diseases from unlikely sources. Walters attributes the rise of Lyme disease to fragmented forests. Dissected by roads and separated by developments, eastern woodlands can no longer sustain large natural predators, but they remain ideal habitats for deer and mice, which can expose humans to ticks carrying the dangerous Lyme spirochete. An ocean away, as forays into sub-Saharan Africa tempted settlers to add bush meat to their sparse diets, HIV made the species jump, Walters suggests, propelling a worldwide AIDS pandemic.

Beyond our abuse of nature, Walters cites antimicrobial misuse as a precipitator of frightening disease. He focuses most on antimicrobial agents in animal feed, accusing policymakers of ignoring the threat of antimicrobial resistance, fearing more the resistance of agricultural interests bent on nurturing their flocks with medicated rations.

The foundation for the author’s discussion varies from rock-solid to rickety, but his half-dozen arguments portray a society more absorbed in immediate gratification than in ultimate consequence. With that, he offers a guarded prognosis that depends on both our cleverness at finding new cures and our commitment to restoring ecologic wholeness.

